# Non-autonomous zinc–methylimidazole oscillator and the formation of layered precipitation structures in a hydrogel

**DOI:** 10.1038/s41598-023-37954-9

**Published:** 2023-07-07

**Authors:** Norbert Német, Hugh Shearer Lawson, Gábor Holló, Nadia Valletti, Federico Rossi, Gábor Schuszter, István Lagzi

**Affiliations:** 1 Department of Physics, Institute of Physics, Budapest University of Technology and Economics, Műegyetem Rkp. 3, Budapest, 1111 Hungary; 2 ELKH-BME Condensed Matter Research Group, Budapest University of Technology and Economics, Műegyetem Rkp. 3, Budapest, 1111 Hungary; 3 Department of Earth, Environmental and Physical Sciences, University of Siena, Pian Dei Mantellini 44, 53100 Siena, Italy; 4 Department of Physical Chemistry and Materials Science, University of Szeged, Rerrich Béla Tér 1, Szeged, 6720 Hungary

**Keywords:** Chemistry, Materials science

## Abstract

Oscillations are one of the intrinsic features of many animate and inanimate systems. The oscillations manifest in the temporal periodic change of one or several physical quantities describing the systems. In chemistry and biology, this physical quantity is the concentration of the chemical species. In most chemical oscillatory systems operating in batch or open reactors, the oscillations persist because of the sophisticated chemical reaction networks incorporating autocatalysis and negative feedback. However, similar oscillations can be generated by periodically changing the environment providing non-autonomous oscillatory systems. Here we present a new strategy for designing a non-autonomous chemical oscillatory system for the zinc–methylimidazole. The oscillations manifested in the periodic change of the turbidity utilizing the precipitation reaction between the zinc ions and 2-methylimidazole (2-met) followed by a partial dissolution of the formed precipitate due to a synergetic effect governed by the ratio of the 2-met in the system. Extending our idea spatiotemporally, we also show that these precipitation and dissolution phenomena can be utilized to create layered precipitation structures in a solid agarose hydrogel.

## Introduction

Oscillators are systems in which at least one of the physical quantities characterizing the system exhibits periodic temporal change. Chemical oscillators are chemical kinetic systems in which the concentrations of the chemical species show oscillatory behavior. Oscillations in these systems can be maintained and manifested either in batch (e.g., Belousov–Zhabotinsky reaction^1–3^, Briggs–Rauscher reaction^4–6^) or open systems^7–11^. Experiments in open systems have been realized in continuous stirred-tank reactors (CSTRs), which contribute to maintaining the chemical systems far from their thermodynamic equilibria. In the past, the exclusively used design strategy to generate chemical oscillations was to couple autocatalytic reactions with negative feedback reactions. Following this strategy, various autonomous oscillatory systems have been designed. Recently, it has been presented that non-autonomous pH oscillations can be engineered and maintained in a simple acid–base neutralization reaction in a CSTR applying temporal inflow functions of the acidic and alkaline solutions in antiphase^12^.

In the past decades, several chemical oscillating systems have been presented comprising inorganic precipitates^13–15^, nanoparticles^16–20^, gels^21^, and fatty acids^22^ having autonomous oscillatory behavior. In these systems, the core oscillator (mostly pH oscillators) has been coupled to these pH-sensitive entities. The generated periodic pH change was translated into the periodic precipitation/complex formation and protonation/deprotonation phenomena causing precipitation and its dissolution, aggregation/disaggregation, or vesicle/micelle transition.

Metal–organic frameworks (MOFs) are a class of inorganic–organic hybrid crystalline coordination polymers known for the past three decades^23^. These materials have high porosity structure and thermal stability, in some cases, they are similar to zeolites (like the zeolitic imidazolate frameworks (ZIFs)^24^. These ordered networks consist of metal ions (mostly copper, zinc, cadmium, and other transition metals) linked together by organic linkers. According to the starting materials, their initial concentrations and ratio, temperature, and reaction time can be used to design structures with different particle sizes, active surfaces, and robustness. In some cases, the pH also has an important role in the organic linker protonation/deprotonation and coordination with the metal ion thus driving the crystallization^25^. MOFs have been used in various applications due to their high porosity such as gas storage and separation^26^, gas and liquid chromatography^27^, drug delivery^28^, and catalyst in organic reactions^29^. They have been also applied in electronics as semiconductors^30^.

Here we present a zinc–methylimidazole (the components of ZIF-8) oscillator – manifesting in the oscillation of the turbidity – utilizing the precipitation reaction between the zinc ions and 2-met followed by a partial dissolution of the formed precipitate due to a synergetic effect governed by the ratio of the 2-met in the system. Extending our idea, we also show that these subsequent phenomena can be utilized to create layered precipitation structures in a solid agarose hydrogel.

## Results

**Figure 1:**
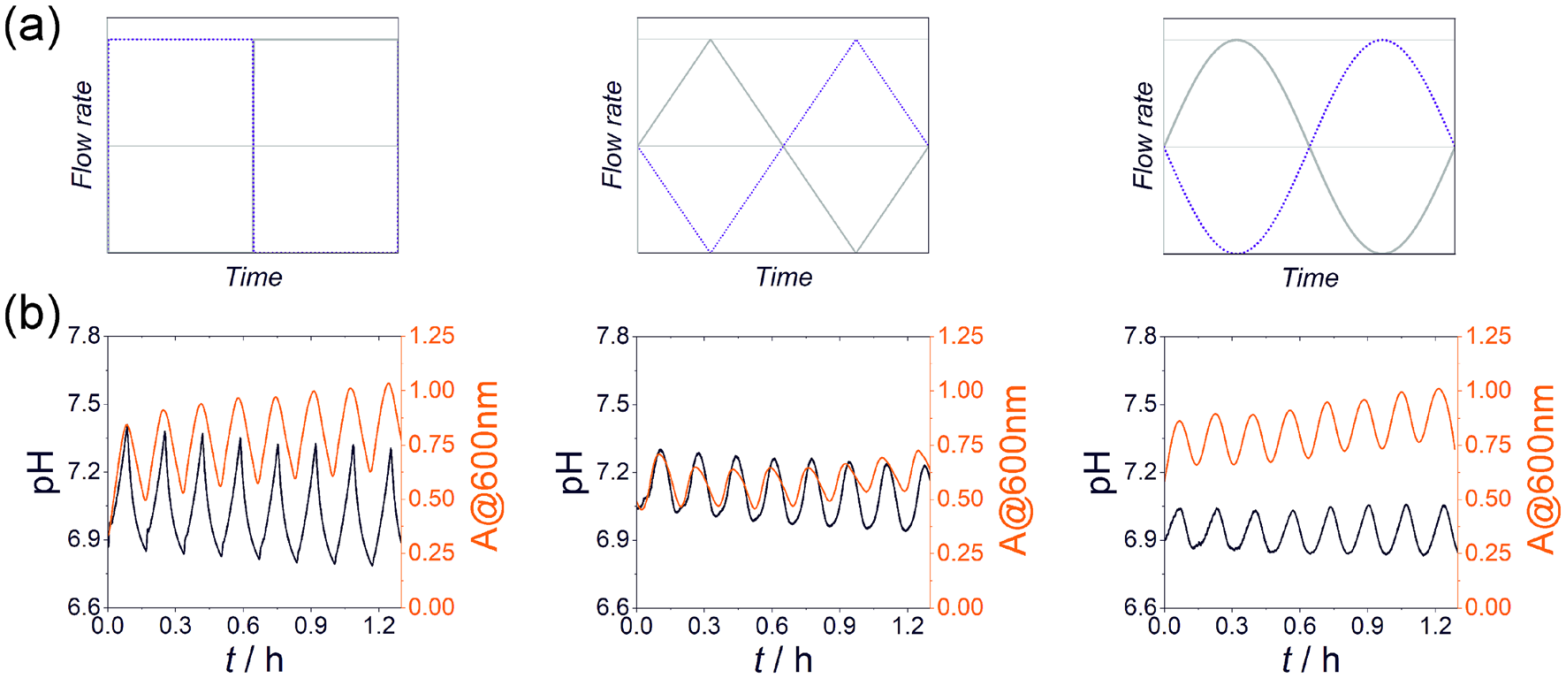
Various time-dependent inflow rate functions (square, triangular, and sinusoidal) in an antiphase condition (marked as solid and dotted curves) for the solutions of the reagents used in the experiments (the temporal phase difference between the two wavefronts is π) (**a**). The generated oscillations in the turbidity and the monitored pH changes in the experiments with the time period of 10 min (**b**). The concentrations of the zinc sulfate and 2-met solutions in the input feed were 20 mM. ${r}_{0}$ (non-modulated flow rate), ${r}_{\mathrm{max}}$ (maximum flow rate) and ${r}_{\mathrm{min}}$ (minimum flow rate) were 15, 29.5, and 0.5 µL s^−1^, respectively. *t* = 0 corresponds to the time when the constant amplitude oscillations appeared.

In a typical experiment, the solutions of the reagents of ZIF-8 were simultaneously introduced into the CSTR, which was initially filled with distilled water, with the modulated flow rate in antiphase in time using two programmable syringe pumps (Supplementary Fig.S1, Supporting Information). The CSTR was a quartz cuvette placed in a UV–vis spectrophotometer. The formation of the zinc–methylimidazole precipitate was monitored by recording the turbidity of the solution. It should be noted that turbidity is affected not only by the concentration but also by the average size, morphology, and size distribution of sol particles. We applied the three most used waveform functions: square, triangular, and sinusoidal waveforms of the inflow rates of the solutions of reagents. One of the syringes contained a solution of zinc ions (20 mM), while the second one had a solution of 2-met (20 mM). The antiphase flow rate in time generated a series of switching between two conditions, one with an excess of the metal ions and the other with an excess of the linker. This switch is also accompanied by a change in the pH. Figure 1 shows the waveforms used for the inflow rates of the reagents and the generated oscillations of the turbidity of the zinc–methylimidazole solutions and pH in the CSTR. In all cases, the turbidity oscillations have sinusoidal shapes with a few tenths of a pH unit irrespectively to the applied wavefront functions. It can be also seen that the non-autonomous oscillatory system had an identical time period compared to the time period of the periodic inflow rate functions (Fig. 1b). We could tune the shape of the pH change with the inflow waveform used. In addition, the shape of the wavefronts influenced the turbidity change as well. When the pH change was sharp due to the instantaneously changed inflow rates (square wavefront) of the reagents, the turbidity change was large as well (Fig. 1a). When pH change was smoother due to the continuous change in the inflow rates (triangular and sinusoidal wavefronts, Fig. 1b, second and third panels), the turbidity oscillations had smaller amplitudes.

Figure 1 presents pH and turbidity curves recorded once the constant amplitude oscillations appeared. Supplementary Fig S2 shows corresponding curves measured from the beginning of the experiments. In all cases, we can observe an apparent induction period. This induction period is because in experiments the solutions of the reagents were simultaneously introduced into the CSTR filled initially with distilled water. At the beginning of the experiments, the concentrations of the reagents in the CSTR were low due to dilution. The concentrations of the reagents increased gradually involving an increase in the turbidity without oscillatory behavior. However, after some time (~ 0.3 h), their concentrations reached the operational level dictated by the pumps, and the pH and turbidity exhibited oscillations in time.

We carried out experiments with zinc acetate as well and could reproduce similar results for the oscillation of the turbidity in time (Supplementary Fig. S3). We performed such a control experiment because the anions of the reagent salts can affect the crystal morphology^31^.

**Figure 2:**
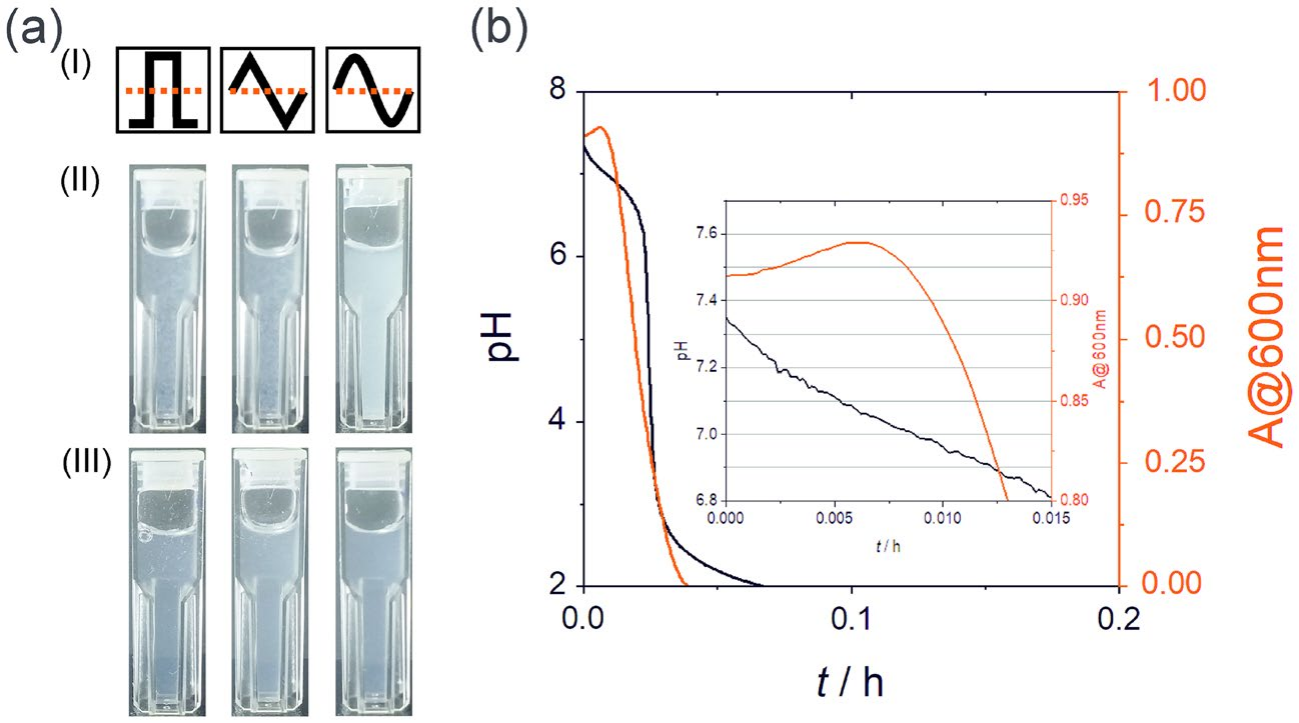
Optical photographs of the contents of the reactors at maximum (II) and minimum (III) pH using various time-dependent inflow rate functions (I) for the solutions of reagents in an antiphase condition (**a**). Titration curve of the zinc–methylimidazole sol (turbidity and pH of the sample versus time) generated by the reaction of zinc sulfate (26.5 mM) and 2-met (73 mM) (**b**). The flow rate of the acidic solution was 30 µL s^−1^. The inset in panel (**b**) shows the enlarged part of the titration curve at the beginning of the titration.

This non-autonomous periodic turbidity change can be explained by the following arguments. First, since the two inflow rates were in antiphase, at the stage when 2-met was in excess in the reactor, it favored the formation of the zinc–methylimidazole precipitate. It caused an increase in the turbidity of the reactor. However, when the zinc ions were in excess, the formation of the zinc–methylimidazole precipitate was suppressed causing the decrease of the turbidity in the CSTR. It is a well-known experimental observation in an aqueous environment that the formation of ZIF-8 requires an excess of 2-met, which favors the formation of the charged intermediate complex. We performed some control experiments showing explicitly that the excess 2-met facilitates the formation of the zinc–methylimidazole precipitate. We carried out experiments in a batch system (cuvette), in which the greatest and lowest concentration ratios for 2-met and zinc ions were created that existed in the reactor for all wavefront functions (Fig. 2a). The maximum and minimum concentration ratios and the corresponding absolute concentrations of the reagents were calculated by using a mathematical model for the CSTR (for the details see the Supporting Information). One can see that the turbidity of the solutions in all cases for the excess 2-met is greater than that of the excess zinc ions. Secondly, the excess 2-met and zinc ions increased and decreased the pH in the reactor (ΔpH ~ 0.3), respectively (Fig. 1), because the 2-met and zinc sulfate solutions are slightly alkaline and slightly acidic, respectively. We found in a control experiment that the decrease in the pH caused the partial dissolution of the formed precipitate. Figure 2b shows the titration curve for the generated zinc–methylimidazole suspension with a solution of hydrochloric acid. In the operational pH window of the oscillator (between 6.7 and 7.5), the turbidity of the solution was significantly decreased. In other words, the excess of zinc ions in the CSTR created a condition for partial dissolution of the formed zinc–methylimidazole precipitate. These two phenomena worked synergistically; the excess of 2-met facilitated the formation of the zinc–methylimidazole precipitate in the CSTR increasing the turbidity. However, the excess zinc ions created the condition of suppressing the precipitation and the decreased pH caused the partial dissolution of the formed zinc–methylimidazole precipitate. Analyzing the titration curve (Fig. 2b), we can observe that the pH change is slow at the beginning of the titration. It can be because Zn^2+^/ZIF-8 or the ZIF-8/2-met are acid/base pairs that act as buffers.

**Figure 3:**
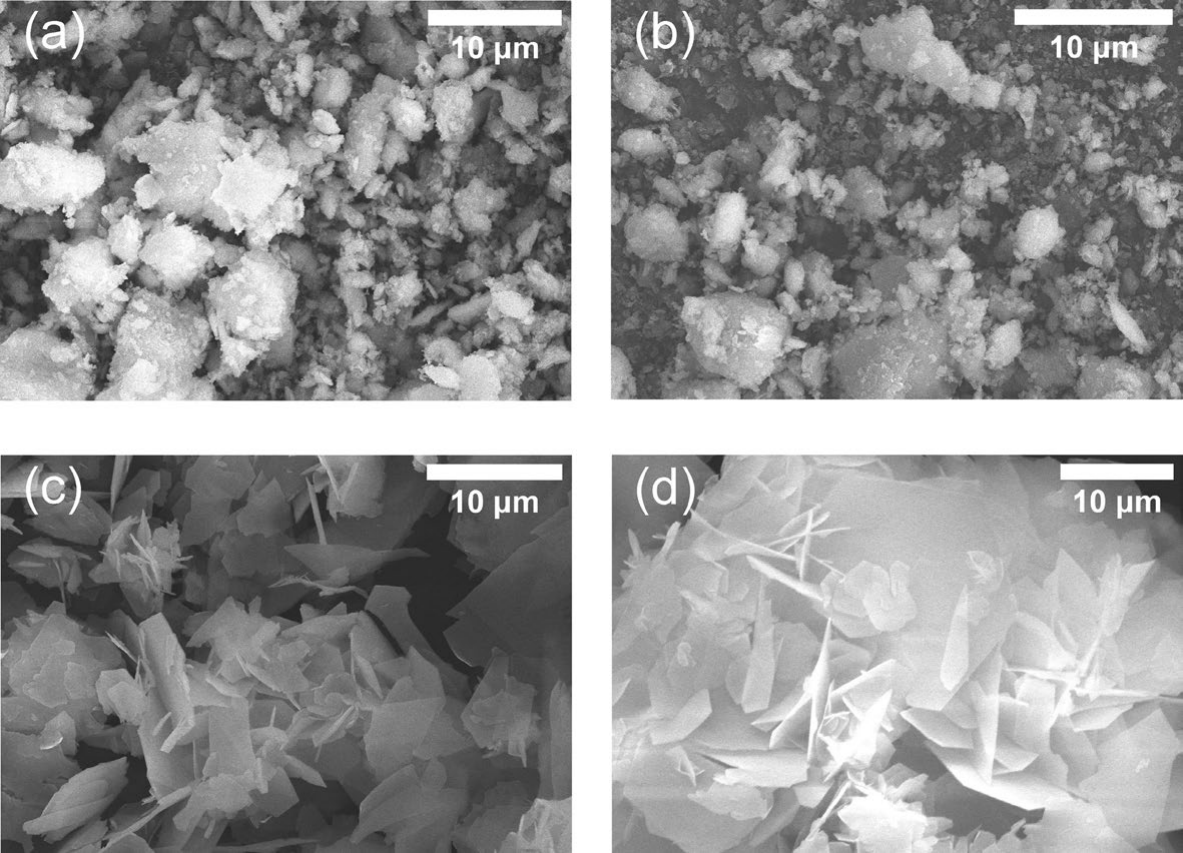
Scanning electron micrographs of the samples collected from the continuous stirred-tank reactor at maximum (**a**, **c**) and minimum (**b**, **d**) pH generated in the reactor using various time periods of the input feeds in an antiphase condition applying sinusoidal modulation: *T* = 10 min (**a**, **b**) and *T* = 3 h (**c**, **d**). The concentrations of the zinc sulfate and 2-met solutions in the input feed were 20 mM. ${r}_{0}$ (non-modulated flow rate), ${r}_{\mathrm{max}}$ (maximum flow rate) and ${r}_{\mathrm{min}}$ (minimum flow rate) were 15, 29.5, and 0.5 µL s^−1^ (**a**, **b**) and 1, 1.9, and 0.1 µL s^−1^ (**c**, **d**), respectively.

In the typical experiments, the time period of the flow rate was 10 min, and the residence time in the CSTR was 4.7 min calculated from the experimental conditions as *t*_res_ = *V*_CSTR_/*r*_nm_, where *r*_nm_ and *V*_CSTR_ are the non-modulated flow rate (through two channels) and the volume of the reactor, respectively. This time is significantly shorter than the usual synthesis time of ZIF-8 (it is usually ~ 24 h)^32^. Scanning electron microscopy (SEM) measurements confirmed that the formed particles have amorphous morphology (Fig. 3a,b). However, once we increased the residence time of the reagents to 70.8 min in the CSTR by reducing the average flow rate (to 2 μL s^−1^) and the time period of the flow rate to 3 h, we could observe different crystal morphology of zinc–methylimidazole precipitate (Fig. 3c,d). The crystals have lamellar structure, and we hypothesize the formation of the *dia* (diamond-like) polymorph of zinc–methylimidazole precipitate having nonporous structures with a significantly lower internal surface area (~ 10 m^2^/g) compared to ZIF-8 (~ 1500 m^2^/g). This polymorph is thermodynamically more stable than ZIF-8, and therefore, it is a common observation in the synthesis of ZIF-8 in the aqueous environment that it may gradually transform into the thermodynamically more stable polymorphs^32^.

**Figure 4:**
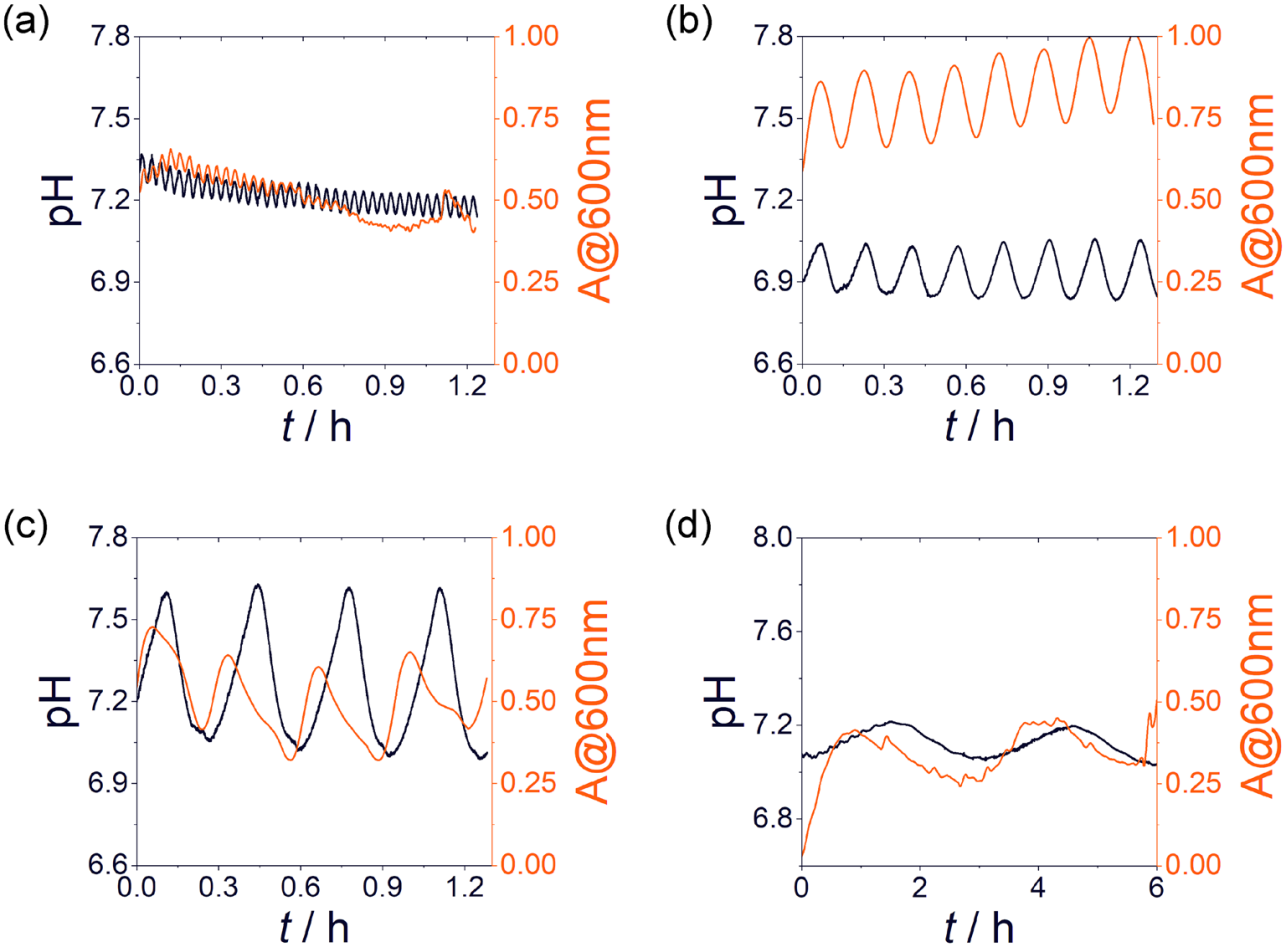
Generated oscillations in the turbidity and the monitored pH changes in the experiments using sinusoidal modulation with various time periods of the input feeds in an antiphase condition, *T* = 2 min (**a**), *T* = 10 min (**b**), *T* = 20 min (**c**), and *T* = 3 h (**d**). The concentrations of the zinc sulfate and 2-met solutions in the input feed were 20 mM. ${r}_{0}$ (non-modulated flow rate), ${r}_{\mathrm{max}}$ (maximum flow rate) and ${r}_{\mathrm{min}}$ (minimum flow rate) were 15, 29.5, and 0.5 µL s^−1^, and in the case of (**d**) were 1, 1.9, and 0.1 µL s^−1^. *t* = 0 corresponds to the time when the constant amplitude oscillations appeared.

To investigate the robustness of the non-autonomous oscillatory system, we performed experiments with various time periods of the flow rate of the reagents ranging from 2 to 45 min using sinusoidal modulation (Fig. 4a-c and Supplementary Fig. S4). We could observe that the increase in the time period of the inflow rate resulted in an increase in the amplitude of both pH and turbidity oscillations with a saturation trend (Supplementary Fig. S5). In experiments, the residence time remained the same, however, greater time periods allowed to maintain high excess states of one of the reagents for a longer time in the CSTR. It should be noted that the experiments with the time period of 3 h (Fig. 4d) cannot be compared to the experiments with the shorter time periods (Fig. 4a-c and Supplementary Fig. S4) because in the case of 3 h, the non-modulated, maximum, and minimum flow rates were lower. We can also notice that there is a phase difference between the pH and turbidity oscillations, which can be due to that precipitation and dissolution are not instantaneous processes.

**Figure 5:**
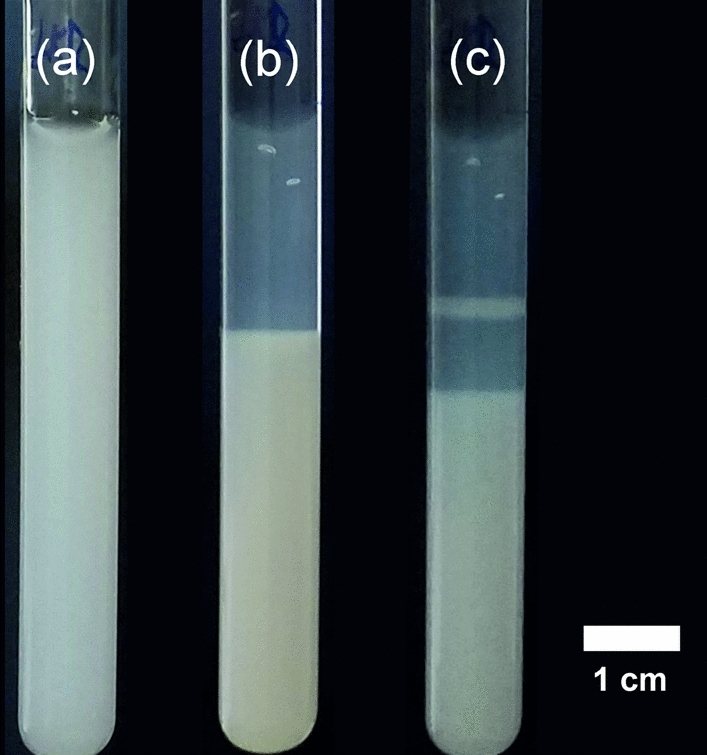
Striped precipitation pattern formed in a test tube filled with zinc–methylimidazole precipitate in a 1% w/w agarose gel. White and transparent regions correspond to the zones of the zinc–methylimidazole precipitate and its dissolved form, respectively. The solution on top of the gel was periodically changed from acid ([HCl] = 0.1 M) to 2-met ([2-met] = 0.1 M) at *t* = 0  (**a**), *t* = 24 h (**b**) and *t* = 44 h (**c**).

To extend this idea spatiotemporally, we carried out experiments in test tubes having zinc–methylimidazole precipitate homogeneously distributed in a hydrogel matrix. To design layered structures of the precipitate, after the gelation process, a solution of hydrochloric acid was layered on top of the solid hydrogel column. Once the acid diffused into the gel, it decreased the pH causing a full dissolution of the precipitate (Fig. 5). After 24 h, the acidic solution was removed and replaced by the solution of 2-met, which diffused in the gel and increased the pH and contributed to the formation of the zone of the zinc–methylimidazole precipitate in the gel column after another 20 h. In this manner, we could achieve a periodic distribution of the precipitate (Fig. 5). We did not observe precipitate near the liquid–gel interface probably because after the dissolution of the ZIF-8, most of the zinc ions diffused to the acidic solution layered above the gel column decreasing its concentration in the gel. It should be noted that in our previous work, we produced periodic precipitation of ZIF-8 and ZIF-67 in a solid agarose gel by separating initially the reagents of ZIFs^34^.

## Discussion

In this study, we introduced a novel concept to design non-autonomous precipitation oscillations. The chemical system was the zinc ions/2-met system, whose components are the regents of ZIF-8. In our setup, the zinc–methylimidazole precipitate was generated in a CSTR fed simultaneously with the modulated flow rate of the solutions of the reagents in antiphase using two programmable syringe pumps. Due to the antiphase feeding, the turbidity and pH exhibited oscillatory behavior in the CSTR. Turbidity oscillations were generated due to a synergetic effect governed by a ratio of the 2-met in the system. Namely, the excess of 2-met created higher pH, and both, higher pH and excess of 2-met, facilitated the formation of the zinc–methylimidazole precipitate generating greater turbidity than in the case of lower pH and excess Zn^2+^. We found that the shape of the waveform of the inflow rate can be used to enhance the amplitude in the pH and turbidity oscillations. Moreover, longer residence time in the CSTR can contribute to the formation of different morphology of the crystals formed in the process. The proposed method can be potentially used in designing and (re)shaping crystals due to the periodically changed environment^33^.

## Methods

### Experimental setup

#### Non-autonomous zinc–methylimidazole oscillators

In our experiments, the formation of zinc–methylimidazole precipitate was carried out in a CSTR (stirring rate of 600 rpm at 21.0 ± 0.5 °C). The CSTR was a quartz cuvette (*V* = 15 mL) with an optical length of 2 cm and placed in a UV–vis spectrophotometer. The constant volume in the cuvette was 8.5 mL. The following reagent-grade chemicals were used in the experiments: zinc sulfate heptahydrate, zinc acetate dihydrate (Merck, Sigma Aldrich), and 2-methylimidazole (Merck, Sigma Aldrich). Two solutions of zinc ions (concentration of the stock solution was 20 mM) and 2-met (concentration of the stock solution was 20 mM) were flowed simultaneously with the modulated flow rate in antiphase into the CSTR using two programmable syringe pumps. Three types of inflow rate functions were used: square, triangular, and sinusoidal waves. The corresponding functions were the following (Fig. 1a):

square function:

1 $$r\left( t \right) = \left\{ {\begin{array}{*{20}l} {r_{{{\text{max}}}} ,} \hfill & {\textrm{if}\; 0 \le t < T/2} \hfill \\ {r_{{{\text{min}}}} , } \hfill & {\textrm{if} \;T/2 \le t < T} \hfill \\ \end{array} } \right.$$

triangular function:

2 $$r\left( t \right) = \left\{ {\begin{array}{*{20}l} {\frac{{r_{{{\text{max}}}} - r_{{{\text{min}}}} }}{2} + \frac{{r_{{{\text{max}}}} - r_{{{\text{min}}}} }}{T/2}t,} \hfill & {\textrm{if} \;0 \le t < \frac{T}{4}} \hfill \\ {\left( {r_{{{\text{max}}}} + \frac{{r_{{{\text{max}}}} - r_{{{\text{min}}}} }}{2}} \right) - \frac{{r_{{{\text{max}}}} - r_{{{\text{min}}}} }}{T/2}t,} \hfill & {\textrm{if}\;\frac{T}{4} \le t < \frac{3T}{4}} \hfill \\ {\left( {r_{{{\text{min}}}} - r_{{{\text{max}}}} - \frac{{r_{{{\text{max}}}} - r_{{{\text{min}}}} }}{2}} \right) + \frac{{r_{{{\text{max}}}} - r_{{{\text{min}}}} }}{T/2}t,} \hfill & {\textrm{if}\;\frac{3T}{4} \le t < T} \hfill \\ \end{array} } \right.$$

sinusoidal function:

3 $$r\left( t \right) = { }r_{0} + r_{{\text{A}}} {\text{sin}}\left( {\frac{2\pi }{T}t} \right)$$

where *T* is the time period of the wave functions. ${r}_{0}$, ${r}_{\mathrm{max}}$ and ${r}_{\mathrm{min}}$ are the non-modulated, maximum, and minimum flow rates, respectively. ${r}_{\mathrm{A}}$ is the amplitude of the modulation in case of the sinusoidal function. In the experiments, the phase difference between the wave functions (*φ*) was set to π corresponding to the antiphase setup. The turbidity and pH in CSTR were monitored by a UV-1600PC (VWR) spectrophotometer using kinetic mode (at *λ* = 600 nm) and a calibrated pH electrode (Mettler Toledo Lab pH Electrode LE422), respectively.

#### Formation of layered zinc–methylimidazole precipitate in a hydrogel

First, we prepared the suspension of the zinc–methylimidazole precipitate by mixing 10 mM of zinc sulfate and 10 mM of 2-met solution in 1:10 ratio. After 1 h, an 1% w/w agarose gel (Type II, Sigma-Aldrich) was prepared by mixing agarose powder in distilled water, and the mixture was heated to 80 °C until the agarose was completely dissolved. The hot solution was poured into the test tubes (with the length and inner diameter of 6.5 cm and 1 cm, respectively) filling two third of the total volume. After the gelation process (25 h), 0.1 M hydrochloric acid and 0.1 M 2-methylimidazole solutions were periodically changed on top of the gel column.

## Supplementary Information

Supplementary Information.

## Data Availability

The datasets used and/or analyzed during the current study available from the corresponding author on reasonable request.
