# Supplementary material for: Non-autonomous zinc–methylimidazole oscillator and the formation of layered precipitation structures in a hydrogel
Source: Sci Rep. 2023 Jul 7;13:11029. doi: 10.1038/s41598-023-37954-9 (PMC10329012; doi:10.1038/s41598-023-37954-9)
Supplement: Supplementary file 1 — Supplementary Information. [file 41598_2023_37954_MOESM1_ESM.pdf]

Supplementary Information

Non-autonomous zinc–methylimidazole oscillator and the formation of layered precipitation structures in a hydrogel

Norbert Német[a], *Hugh Shearer Lawson*[a], *Gábor Holló*[b], *Nadia Valletti*[c], *Federico Rossi*[c], *Gábor Schuszter*[d], *István Lagzi*[a,b]*

[a]Department of Physics, Institute of Physics, Budapest University of Technology and Economics, Műegyetem rkp. 3, H-1111 Budapest, Hungary

[b]ELKH-BME Condensed Matter Research Group, Budapest University of Technology and Economics, Műegyetem rkp. 3, H-1111 Budapest, Hungary

[c]Department of Earth, Environmental and Physical Sciences, University of Siena, Pian dei Mantellini 44, 53100 Siena, Italy

[d]Department of Physical Chemistry and Materials Science, University of Szeged, Rerrich Béla tér 1, H-6720 Szeged, Hungary

Numerical model

To estimate the possible maximum and minimum concentrations of the reagents which could occur - in the continuous stirred-tank reactor (CSTR) - without any chemical reactions, a simple model was developed. The concentration change only occurs due to the modulated inflow rates. The system can be described by the following set of differential equations:

$$\frac{\mathrm{d}c_{\mathrm{Zn}^{2+}}}{\mathrm{d}t} = k_{\mathrm{Zn}^{2+}} c^{*}_{\mathrm{Zn}^{2+}} - \kappa c_{\mathrm{Zn}^{2+}}, \tag{S1}$$

$$\frac{\mathrm{d}c_{\mathrm{HMIM}}}{\mathrm{d}t} = k_{\mathrm{HMIM}} c^{*}_{\mathrm{HMIM}} - \kappa c_{\mathrm{HMIM}}, \tag{S2}$$

where $c_{\mathrm{Zn}^{2+}}$ and c_{HMIM} are concentrations of zinc ion and 2-methylimidazole in the CSTR, $c^{*}_{\mathrm{Zn}^{2+}}$ = 20 mM and c^{*}_{HMIM} = 20 mM are the inflow concentrations; $k_{\mathrm{Zn}^{2+}}$ and k_{HMIM} are the inflow rates of the zinc ion and 2-methylimidazole. κ is the outflow rate of the components calculated as $\kappa = k_{\mathrm{Zn}^{2+}} + k_{\mathrm{HMIM}}$.

In the case of a sinusoidal waveform, we used the following inflow rates:

$$k_{\mathrm{Zn}^{2+}} = k_0 + k_{\mathrm{A}} \sin\left(\frac{2\pi}{T} t\right), \tag{S3}$$

$$k_{\mathrm{HMIM}} = k_0 + k_{\mathrm{A}} \sin\left(\frac{2\pi}{T} t + \pi\right), \tag{S4}$$

where $k_0 = r_0/V_{\mathrm{CSTR}}$ and $k_{\mathrm{A}} = r_{\mathrm{A}}/V_{\mathrm{CSTR}}$ are space velocity constants (reciprocal average residence time) and T is the time period of the sinusoidal modulation. r_0, r_{A} and V_{CSTR} are the non-modulated flow rate, the amplitude of the modulated flow rate, and the volume of the CSTR, respectively. The ordinary differential equations were solved using MATLAB software. The initial conditions were the following, $c^{0}_{\mathrm{Zn}^{2+}} = c^{0}_{\mathrm{HMIM}} = 0$, $r_0 = 15\ \mu\mathrm{L\ s}^{-1}$, while $r_{\mathrm{A}} = 14.5\ \mu\mathrm{L\ s}^{-1}$, T = 600 s, and V_{CSTR}= 8.5 mL.

In simulations, we calculated the maximum and minimum concentrations of the reagents in the CSTR for each type of waveform in a similar manner (for the functions and parameters see the main text the section "Methods") (**Table S1**).

Table S1. The concentration of the ZIF-8 material components at the extreme values of the oscillation in each type of waveform.

**Waveform**	**Maximum c_{HMIM} (mM)/ Minimum $c_{\mathrm{Zn}^{2+}}$ (mM)**	**Maximum $c_{\mathrm{Zn}^{2+}}$ (mM)/ Minimum c_{HMIM} (mM)**
Square	14.69/5.31	
Triangular	12.45/7.55	
Sinusoidal	13.00/7.00	

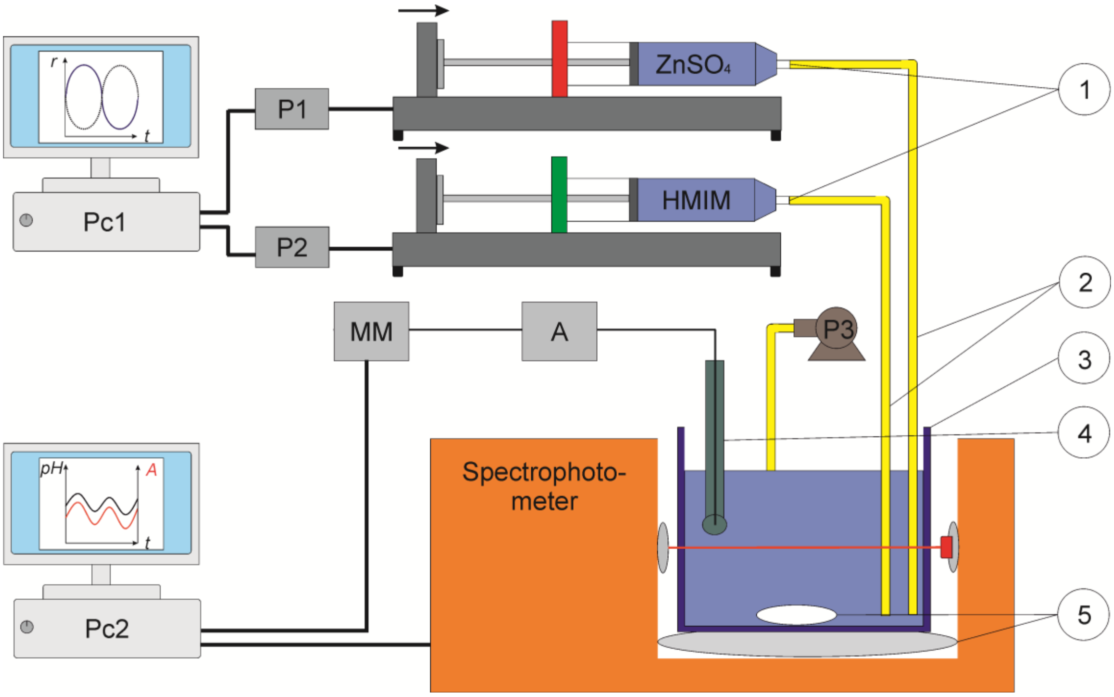

Supplementary Fig. 1 The sketch of the experimental setup. Syringes filled with Zn^{2+} and 2-met stock solutions (1) are placed in programable syringe pumps (P1, P2). The stock solutions are pumped through Tygon tubes (2) into a quartz cuvette (volume of V = 14 mL and optical length of l = 2 cm; the constant volume in the cuvette was 8.5 mL) (3) placed in a UV-vis spectrophotometer. pH changing was measured in real-time with a calibrated glass electrode (4) connected to an amplifier (A) and a multimeter (MM). The volume of the reaction mixture was kept constant with a peristaltic pump (P3) and stirred with a magnetic stirrer (5). Pc1-Pc2 are computers.

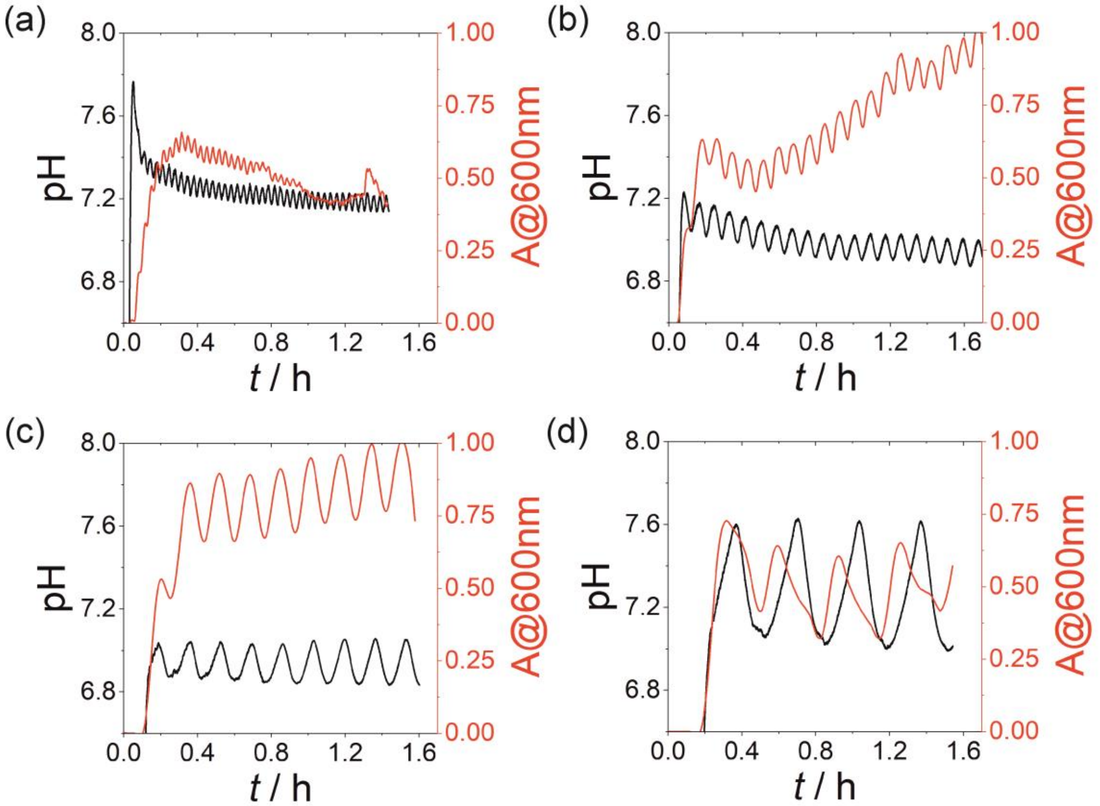

Supplementary Fig. 2 Generated oscillations in the turbidity and the measured pH changes monitored from the beginning of the experiments using sinusoidal modulation with various time periods of the input feeds in an antiphase condition, T = 2 min (a), T = 5 min (b), T = 10 min (c), and T = 20 min (d). The concentrations of the zinc sulfate and 2-met solutions in the input feed were 20 mM. r_0 (non-modulated flow rate), $r_{\max}$ (maximum flow rate) and $r_{\min}$ (minimum flow rate) were 15, 29.5, and 0.5 μL s^{-1}, respectively.

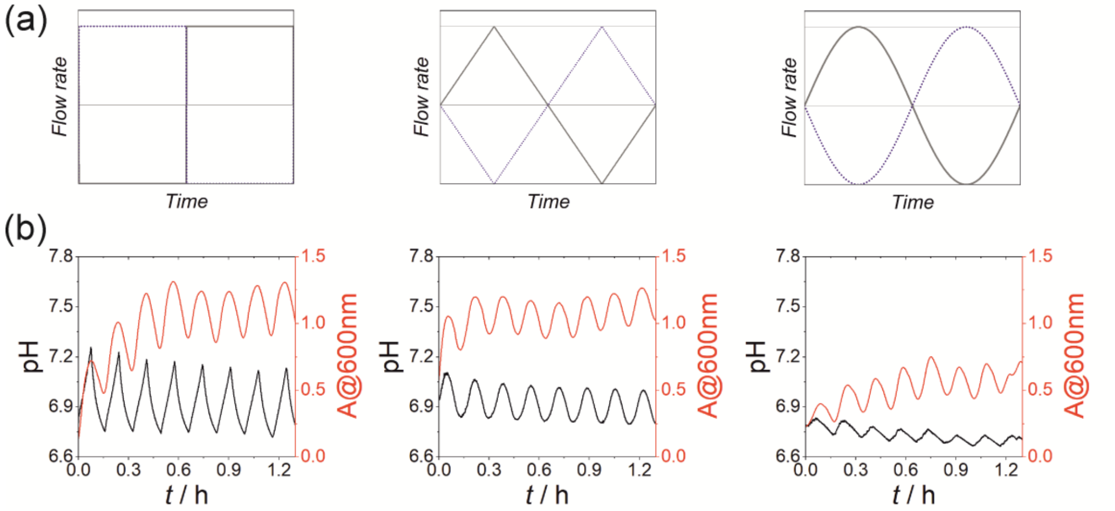

Supplementary Fig. 3 Various time-dependent inflow rate functions (square, triangular, and sinusoidal) in antiphase condition (the temporal phase difference between the two wavefronts is π) used in the experiments (a). The generated oscillations in the turbidity using zinc acetate and the monitored pH changes in the experiments (b). The concentrations of the zinc acetate and 2-met solutions in the input feed were 20 mM. r_0 (non-modulated flow rate), r_{max} (maximum flow rate) and r_{min} (minimum flow rate) were 15, 29.5, and 0.5 μL s^{-1}, respectively. t = 0 corresponds to the time when the constant amplitude oscillations appeared.

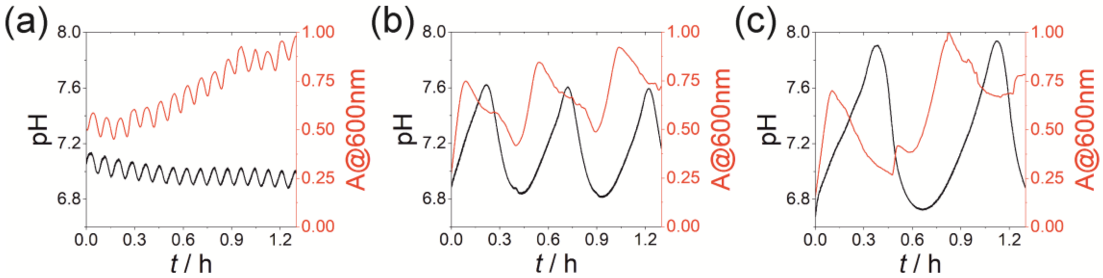

Supplementary Fig. 4 Generated oscillations in the turbidity and the monitored pH changes in the experiments using sinusoidal modulation with various time periods of the input feeds in an antiphase condition, T = 5 min (a), T = 35 min (b), and T = 45 min (c). The concentrations of the zinc sulfate and 2-met solutions in the input feed were 20 mM. r_0 (non-modulated flow rate), $r_{\max}$ (maximum flow rate) and $r_{\min}$ (minimum flow rate) were 15, 29.5, and 0.5 μL s^{-1}, respectively. t = 0 corresponds to the time when the constant amplitude oscillations appeared.

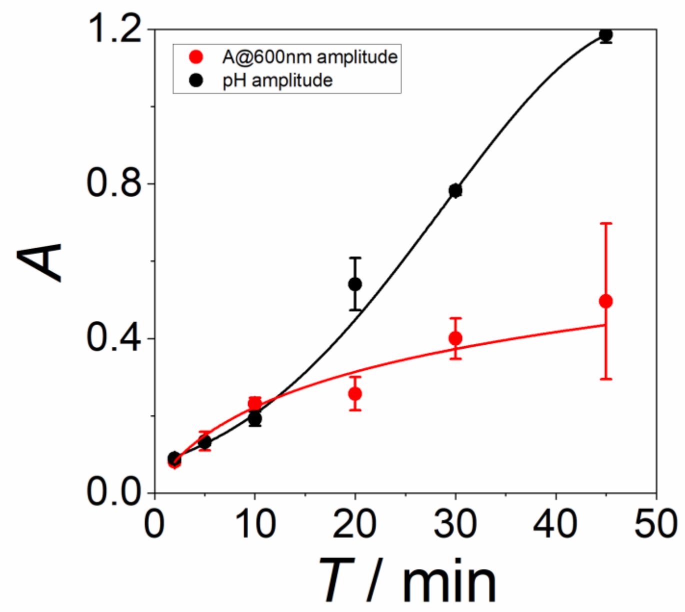

Supplementary Fig. 5 Dependence of the amplitude of the generated pH (black) and turbidity (red) on the time period of the inflow rate of the reagents in the CSTR using sinusoidal modulation. The concentrations of the zinc sulfate and 2-met solutions in the input feed were 20 mM. r_0 (non-modulated flow rate), r_{max} (maximum flow rate) and r_{min} (minimum flow rate) were 15, 29.5, and 0.5 μL s^{-1}, respectively.
